# Hematopoiesis Revolves Around the Primordial Evolutional Rhythm of Purinergic Signaling and Innate Immunity – A Journey to the Developmental Roots

**DOI:** 10.1007/s12015-024-10692-9

**Published:** 2024-02-16

**Authors:** Mariusz Z. Ratajczak, Kamila Bujko, Katarzyna Brzezniakiewicz-Janus, Janina Ratajczak, Magdalena Kucia

**Affiliations:** 1 Laboratory of Regenerative Medicine, Medical University of Warsaw, Warsaw, Poland; 2 Department of Hematology, University of Zielona Gora, Multi-Specialist Hospital Gorzow Wlkp., Gorzow Wielkopolski, Poland; 3 Stem Cell Institute at James Graham Brown Cancer Center, University of Louisville, 500 S. Floyd Street, Rm. 107, Louisville, KY 40202 USA; 4 Center for Preclinical Studies and Technology, Department of Regenerative Medicine at Medical, University of Warsaw, Warsaw, Poland

**Keywords:** Purinergic signaling, Complement, Complosome, Innate immunity, NLRP3 inflammasome, Nox2, ROS, RNS, Stem cell homing and engraftment, Stem cell metabolism, Hematopoiesis

## Abstract

A cell's most significant existential task is to survive by ensuring proper metabolism, avoiding harmful stimuli, and adapting to changing environments. It explains why early evolutionary primordial signals and pathways remained active and regulate cell and tissue integrity. This requires energy supply and a balanced redox state. To meet these requirements, the universal intracellular energy transporter purine nucleotide-adenosine triphosphate (ATP) became an important signaling molecule and precursor of purinergic signaling after being released into extracellular space. Similarly, ancient proteins involved in intracellular metabolism gave rise to the third protein component (C3) of the complement cascade (ComC), a soluble arm of innate immunity. These pathways induce cytosol reactive oxygen (ROS) and reactive nitrogen species (RNS) that regulate the redox state of the cells. While low levels of ROS and RNS promote cell growth and differentiation, supra-physiological concentrations can lead to cell damage by pyroptosis. This balance explains the impact of purinergic signaling and innate immunity on cell metabolism, organogenesis, and tissue development. Subsequently, along with evolution, new regulatory cues emerge in the form of growth factors, cytokines, chemokines, and bioactive lipids. However, their expression is still modulated by both primordial signaling pathways. This review will focus on the data that purinergic signaling and innate immunity carry on their ancient developmental task in hematopoiesis and specification of hematopoietic stem/progenitor cells (HSPCs). Moreover, recent evidence shows both these regulatory pathways operate in a paracrine manner and inside HSPCs at the autocrine level.

**Graphical Abstract:**

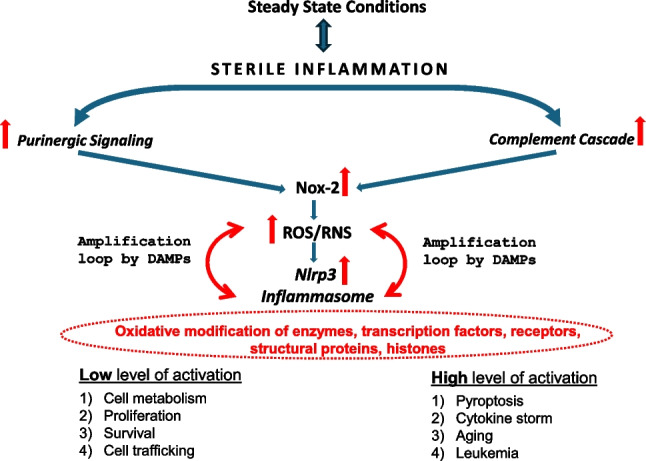

## Introduction

Development and lineage specification of hematopoietic stem/progenitor cells (HSPCs) is regulated by the coordinated action of peptide-based growth factors, cytokines, chemokines, and bioactive lipids [1, 2]. However, all these factors emerged in biological systems later during the evolution. Thus, to understand hematopoiesis better, we must go back in time and look for primordial signaling pathways that originated early at the beginning of evolution and, till today, modulate the functional integrity of the hematopoietic system. These old signaling pathways affect adult hematopoiesis and, in addition, modulate the expression of “classical” lineage-specific hematopoietic factors that emerged during evolution [1–5]. The ancient development regulators directly affect the fate of HSPCs and the function of the hematopoietic microenvironment in bone marrow (BM). Both purinergic and innate immunity signaling regulate the redox state of the cells that must remain in the physiologically beneficial low range because if hyperactivated, it has opposite adverse effects [6–10].

Moreover, both these pathways operate in a paracrine manner and at the autocrine level in HSPCs [8–14]. Herein, we will discuss data that purinergic signaling and innate immunity carry on their ancient developmental potential in hematopoiesis. These pathways modulate the level of reactive oxygen (ROS) and reactive nitrogen species (RNS), which regulate the intracellular redox state of HSPCs to modulate their biology [15–17].

The fate of the single-cell organisms is regulated by intracrine signaling and responds to extracellular stimuli related to danger signaling and nutrient availability. This becomes more complex in multicellular organisms when paracrine interactions gain importance. Thus, it explains why two essential signaling pathways that regulate vital cellular functions originated from the interaction between mediators of energy supply and proteins involved in innate immunity [12]. For example, glucose uptake, metabolism rate, and intracellular redox state may activate important intracellular innate immunity pattern recognition receptor—Nlrp3 inflammasome [18]. This somehow unique receptor is also activated by complement cascade (ComC) cleavage fragments, and, on the other hand, a lack of complement proteins leads to altered metabolism of hematopoietic cells and negatively affects Nlrp3 inflammasome expression [14]. What is important is that this intracellular innate immunity receptor's expression is regulated by the cells' redox state [19].

This review will present evidence that purinergic signaling and innate immunity dictate intracellular redox state balance, regulating normal steady-state and stress hematopoiesis. This coordinated action allows for lineage specification in response to more specific growth factors, cytokines, and chemokines to maintain a constant number of stem cells in BM. During stress hematopoiesis, purinergic signaling and innate immunity respond to non-inflammatory Danger Associated Molecular Patterns (DAMPs) or inflammatory Pathogen Associated Molecular Patterns (PAMPs) cues [20–22]. This allows the enhanced proliferation, migration, and metabolism of HSPCs to meet new challenges [18–23]. From several DAMPs released from activated/damaged cells, adenosine triphosphate (ATP) that initiates purinergic signaling is the earliest and most important member [23]. Recent evidence indicates that both purinergic and ComC signaling operate in a paracrine-dependent manner and are also functional inside cells [8–14, 24]. This expands our knowledge of the autocrine regulation of hematopoiesis [25]. We will present evidence that hematopoiesis revolves continuously around this primordial evolutional “rhythm” of purinergic signaling and innate immunity.

We will focus on the purinergic P2X7 receptor and ComC C5aR1 receptor, which have different molecular structures. The P2X7 receptor is an ion-gated channel that induces Ca^2+^ influx and K^+^ efflux [23, 24], and C5aR1 is implicated in β-arrestin2 recruitment via Rab5a, coupling of G_αi_ proteins, ERK1/2 phosphorylation, calcium mobilization, and Rho activation [26]. However, what is important for this review is that both receptors regulate the redox state of the cells and activate the Nlrp3 inflammasome [19, 27, 28].

### Purinergic Signaling as a Primordial Signaling Pathway in Hematopoiesis

**Fig. 1:**
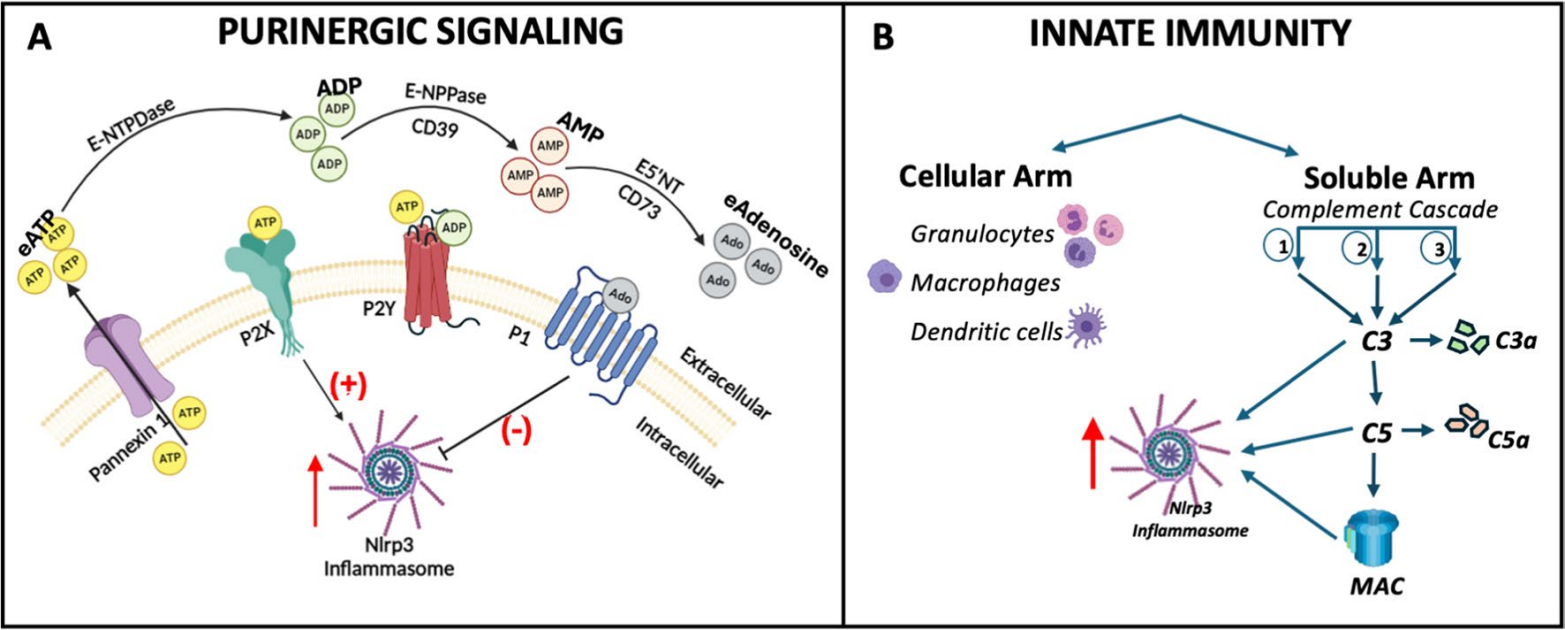
Overview of the elements of purinergic signaling and innate immunity.** Panel A -**ATP secreted from cells becomes in extracellular space important signaling molecule extracellular ATP (**eATP**) and is processed to ADP, AMP, and extracellular adenosine (**eAdo**), mainly by ecto-nucleoside triphosphate diphosphohydrolase (eNTDPase, also known as CD39) and 5-nucleotidase (5'-NT, also known as CD73**)**. Purinergic signaling involves ionotropic P2X, G protein-coupled P2Y, and P1 receptors. P1 receptors are activated by **eAdo**. This scheme has been simplified for clarity. **Panel B**. Innate or natural immunity is present at birth and does not have to be learned through exposure to an invader and provides an immediate response to extrinsic and intrinsic challenging factors and stressors. The cellular arm comprises phagocytes, mast cells, basophils, eosinophils, and dendritic cells. The most important member of the soluble arm is the complement cascade (ComC), which consists of more than 30 proteins that are activated in a sequence to defend against infection. ComC becomes activated by three pathways known as the (1) classical, (2) mannan-binding lectin, and (3) alternative pathway to release active cleavage fragments of C3 and C5 components (C3a and C5a anaphylatoxins), and the C5b-C9 membrane attack complex (MAC). This scheme has been simplified for clarity

As mentioned above, the origin of purinergic signaling is related to cell metabolism and universal energy transfer molecules, such as purine nucleotide-adenosine triphosphate (ATP). This purine is secreted from the activated cells [23], e.g., by pannexin-1 channels, connexin-43 hemichannels, or during exocytosis of microvesicles enriched for this nucleotide (Fig. 1A). Moreover, the loss of plasma membrane integrity by stress mechanisms also leads to the release of ATP [27]. If released from the cytosol into extracellular space, it becomes extracellular ATP (eATP) – a “potent signaling molecule” that interacts with several ionotropic P2X and selected G-protein coupled P2Y purinergic receptors [20, 26]. These receptors are among the most abundant in living organisms and originated a billion years ago, early in evolution. It explains why they are also highly expressed on HSPCs [20].

*- Biology of purinergic signaling.* In 1972, Geoffrey Brunstock ignited decades of skepticism and controversy that intracellular molecular energy transmitter ATP is released from the cells and that eATP is a potent extracellular signaling mediator [27]. Nevertheless, as often in science, after years of prolonged disbelief, purinergic signaling was gradually accepted. Several receptors were cloned, and drugs based on blocking these receptors were developed for clinical applications. Today, purinergic signaling is regarded as a general intercellular communication system of many, if not all, tissues in the body [20]. This primordial signaling is initiated by the release of eATP that, in extracellular space, is metabolized to extracellular adenosine (eAdo) [20, 28, 29]. While eATP stimulates numerous pathways in HSPCs and the hematopoietic microenvironment, eAdo is an anti-inflammatory nucleotide with several opposite effects [20, 28]. The metabolism of eATP to eAdo proceeds by the formation of adenosine diphosphate (ADP) and adenosine monophosphate (AMP) and is mediated by cell surface-expressed ectonucleotidases including, e.g., CD39 and CD73, and by alkaline phosphatase [23, 27, 29]. In addition to eATP and eAdo, there are released from the cells other rare signaling extracellular nucleotides, including some pyrimidines like uridine triphosphate (UTP), uridine diphosphate (UDP), or UDP-glucose [20, 30]. Nevertheless, eATP and eAdo remain the most critical purinergic signaling molecules in hematopoiesis [20, 28].

There are three known purinergic receptor classes: P1, P2X, and P2Y. While P1 and P2Y are G-protein coupled receptors, P2X receptors are ligand-ion-gated channels [23]. The P2X receptor family is stimulated exclusively by eATP and consists of seven members (P2X1, 2, 3, 4, 5, 6, and 7) [20, 31]. The P2Y family consists of eight receptors (P2Y1, 2, 4, 6, 11, 12, 13, and 14) that respond to eATP, eADP, eUTP, eUDP, or eUDP-glucose [23, 29, 31]. Finally, the P1 family comprises four subtypes (A_1_, A_2_A, A_2_B, and A_3_) activated by eAdo [23, 29, 31]. This demonstrates the complexity of this primordial signaling system that evolved with time in multicellular organisms into a remarkable regulatory network orchestrating several biological processes, including hematopoiesis. While P2X and P2Y receptors activate several pathways related to cell migration and proliferation in a positive way, activation of P1 receptors often has the opposite effect.

#### The Role of Purinergic Signaling in HSPCs Trafficking

The role of purinergic signaling in normal hematopoiesis was the subject of some previous studies [30, 32, 33]. Our group, however, became interested in the role of purinergic signaling in the trafficking of HSPCs as occurs during pharmacological mobilization [34, 35], homing, and their engraftment after transplantation [36–39]. To address this issue first, we phenotyped normal human CD34^+^ and murine Sca-1^+^lin^−^CD45^+^ cells enriched for HSPCs for expression of the purinergic receptors and CD39 and CD73 ectonucleotidases involved in the enzymatic conversion of eATP to eAdo [28, 38]. We confirmed the presence of all purinergic receptors and eATP processing enzymes in purified murine and human HSPCs.

In our subsequent functional studies, we focused on P2X ionotropic receptors activated exclusively by eATP expressed on both human and murine HSPCs [28, 34, 36, 37, 40]. Since these receptors are structurally related and expressed simultaneously, we expected to see some potential functional redundancy characteristic of evolutionary old regulatory pathways. We began our work with those P2X receptors that are most abundant on murine HSPCs and employed available P2X4-KO and P2X7-KO animals and, in the case of P2X1 and P2X3 specific receptor inhibitors [28, 34, 36, 37, 40]. We refrained from studying P2X2, P2X5, and P2X6 members, as these receptors have been dammed to be less involved in normal hematopoiesis [20]. We learned that the absence of functional P2X1, P2X3, P2X4, and P2X7 receptors on murine and human HSPC resulted in their defective chemotactic Transwell migration to major BM-homing chemoattractants, including α-chemokine stromal-derived factor-1 (SDF-1), bioactive phosphosphingolipid—sphingosine-1 phosphate (S1P), and eATP [28, 34, 36, 37, 40]. The outcome of these assays indicated that P2X receptors may modulate the HSPCs trafficking as seen in pharmacological mobilization and their homing and engraftment in bone marrow (BM) after transplantation.

Next, we focused on pharmacological mobilization in vivo. We employed the most common pro-mobilizing drugs used in the clinic, such as granulocyte-colony stimulating factor (G-CSF) and CXCR4 receptor antagonist AMD3100 (Plerixafor) to mobilize P2X4-KO, P2X7-KO animals, and wild-type (WT) mice exposed to P2X1 and P2X3 inhibitors [28, 34, 36, 37, 40]. In all these cases, we noticed impaired egress of HSPCs from BM into peripheral blood (PB). Defective mobilization also occurred when we blocked with a small peptide, the Pannex-1 channel, which releases ATP into extracellular space. This confirmed that eATP is an important trigger promoting the mobilization of HSPCs [39]. At the same time, we noticed that exposure of mice to eATP metabolite eAdo has an opposite effect on the mobilization process, supporting a known negative role of eAdo in eATP-mediated processes. To investigate this further, mice exposed to a small molecular inhibitor of CD39 and CD73 cell surface ectonucleotidases processing conversion of eATP to eAdo displayed enhanced mobilization of HSPCs due to a decrease in extracellular eAdo level in BM microenvironment [38]. We found that the inhibitory effect of eAdo was mediated by the A_2_B receptor from the P1 family, and blocking this receptor by a small molecular inhibitor positively impacted the yield of mobilized HSPCs [41]. This observation was subsequently confirmed using A_2_B-KO animals, which, as expected, mobilized better compared to wild-type littermates [41]. Finally, while injection of eATP into mice mobilized by G-CSF or AMD3100 promoted, injection by eAdo inhibited the egress of HSPCs from BM into PB [40]. These observations could have a translational implication in improving clinical mobilization protocols as small molecular inhibitors of A_2_B receptors are available for clinical use.

Moreover, the P2Y receptor family is activated in addition to eATP by several other nucleotides, and one of these receptors P2Y14 is triggered by a rare non-conventional nucleotide UDP-glucose [31]. It has been reported that UDP-glucose preferentially mobilizes long-term repopulating hematopoietic stem cells [30]. Nevertheless, the role of the other members of the P2Y receptor family in HSPCs trafficking needs further detailed investigations.

In the following experiments, BM cells were exposed ex vivo to eATP before infusion into myeloablated recipient mice [37]. This simple procedure enhanced the homing and engraftment of transplanted cells. This established in mice procedure may sensitize human HSPCs before transplantation, allowing them to navigate better BM stem cell niches. In control experiments, incubation with eAdo had the opposite effect [37].

Finally, we asked how important the purinergic signaling network is in the hematopoietic microenvironment. It is known that BM-derived stroma cells, as well as endothelial cells, express purinergic receptors highly [42, 43]. Thus, we tested homing and engraftment of normal wild-type (WT) BM cells transplanted into P2X4-KO and P2X7-KO mice as well as into mice exposed before transplantation to P2X1 and P2X3 small molecular inhibitors [28, 34, 36, 37, 40]. We found that all these animals defective in selected P2X receptor activity display decreased homing and engraftment of transplanted WT BM cells. Interestingly, homing and engraftment were improved if we blocked the level of eAdo in BM by employing CD39 or CD73 ectonucleotidase inhibitors [38]. This effect was subsequently confirmed in A_2_B receptor KO animals [37]. This indicates that while fueled by eATP, sterile inflammation in BM promotes homing and engraftment, eAdo, an anti-inflammatory nucleotide, impairs hematopoietic reconstitution. Finally, experiments with pannexin-1 blocking peptides confirmed additionally the critical role of eATP release from the BM microenvironment in providing the permissive microenvironment for the homing and engraftment of transplanted BM cells [39].

#### The Role of Purinergic Signaling In Stem Cell Metabolism

Purinergic signaling plays an important role in metabolism at the level of food uptake, regulating appetite [44], and at the cellular level, affecting metabolic processes [45]. A crucial potential link to metabolism is the presence of purinergic receptors on the cell surface membranes and, in the case of P2X7, also on mitochondria [24]. Of note, the P2X7 receptor is linked to NADPH oxidase-2 (Nox-2) and, through the release of ROS and RNS, may activate the Nlrp3 inflammasome [46].

The NADPH oxidase (Nox) family includes seven isoforms with different activation mechanisms; however, Nox-2 seems to be associated with hematopoietic cells [47]. The most prominent ROS is superoxide anion radical (O_2_^−^) generated, e.g., by Nox-2 and mitochondrial electron transporter chain [15–17]. Other ROS include hydrogen peroxide (H_2_O_2_) and hydroxyl radicals, and the most critical RNS comprises NO, nitrosonium cation (NO^+^), nitrosonium anion (NO^−^), and peroxynitrite (ONOO^−^) [16, 17]. Low levels of ROS and RNS within the “hormetic zone” benefit cell biology [8–10]. In contrast, at higher levels outside the “hormetic zone,” they are inflicted in cellular aging, diseases, and death [8]. To explain these effects at the molecular level, ROS and RNS may be modified by the oxidation of methionine and cysteine present in enzymes, transcription factors, signaling molecules, and structural proteins. Thus, depending on the level of modification and function of targeted proteins, the biological effects may be different. Moreover, in addition to mitochondria membranes, P2X7 has been reported intracellularly on nuclear cell membranes and lysosomes, which indicates a potential role of ATP in autocrine signaling [48]. In addition to P2X7, more work is needed to evaluate other P2X and P2Y receptors in the metabolism of HSPCs and the role of RNS in cell metabolism in the context of Nlrp3 inflammasome activation [19, 49]. For example, peroxynitrite, an RNS member and strong oxidant generated by the interaction of superoxide with nitric oxide may either promote or inhibit NLRP3 inflammasome activation in non-hematopoietic cells [49]. This, again, may be dose-related. The role of the Nox-2-ROS/RNS-Nlrp3 axis in metabolism will be discussed later.

### The Role of Primordial Innate Immunity Signaling in Hematopoiesis

Innate immunity appeared in evolution more than 1 billion years ago and today comprises the cellular and soluble arm [50]. While the cellular component comprises phagocytes, mast cells, basophils, eosinophils, and dendritic cells, the most important member of the soluble arm is the complement cascade (ComC) [50] Fig. 1B. As mentioned above, some ancient proteins involved in intracellular metabolism likely gave rise to this most abundant ComC member, which is C3 [51].

ComC becomes activated by three pathways known as the i) classical, ii) mannan-binding lectin, and iii) alternative pathway, to release active cleavage fragments of C3 and C5 components (C3a and C5a anaphylatoxins), and finally the C5b-C9 membrane attack complex (MAC) [50]. All three ComC activation pathways respond to danger signals by PAMPs released during infections or DAMPs released upon non-infectious sterile inflammation. Moreover, PAMPs and DAMPs may activate innate immunity receptors expressed on outer cell membranes (e.g., Toll-like receptors, TLRs) or some in the cytosol, including Nlrp3 inflammasome [52]. This should not surprise, as hematopoietic cells are developmentally related to immune cells as they share a common stem cell precursor and thus respond to similar stimuli [53]. It is known that activation of the TLR4 receptor by PAMPs or DAMPs plays an important role in the basic expression level of Nlrp3 inflammasome in innate immunity cells and HSPCs [19, 52].

The primary source of complement proteins circulating in PB is the liver; however, recent evidence indicates that complement proteins are expressed in several types of cells in the body [11, 14, 54, 55]. This intracellular complement, known as “complosome", may regulate several cell functions by engaging cytosol and cell surface-expressed ComC receptors (C3aR, C5aR1, and C5aR2) if activated. The biological effects mediated by intracellular complosome have already been reported for T lymphocytes, monocytes, granulocytes, bone marrow fibroblasts, and some solid tumor cells [11, 14, 54–57]. What seems to be exciting is that we noticed recently complosome expression in HSPCs [14] and several other types of BM-residing stem cells, including mesenchymal stem cells (MSCs), endothelial progenitors (EPCs), and very small embryonic-like-stem cells (VSELs) [58]. This opens a new area of investigation on the role of complosome and innate immunity in regulating the biology of stem cell compartment.

#### The Role Of Liver-Derived Circulating In PB Complement in the Trafficking of HSPCs

Our previous research demonstrated that C5-deficient mice are poor mobilizers in response to G-CSF or Plerixafor [59]. Moreover, C3-KO and C5-KO mice engrafted poorly with transplanted wild-type BM cells [60, 61]. Since C3a and C5a do not chemoattract HSPCs, this data was initially challenging to explain. To solve this issue, we demonstrated that circulating in PB C5a as a potent chemoattractant for granulocytes and promotes the egress of these cells from BM. This step paves the way for HSPCs to follow in their footsteps across the BM-PB barrier during the mobilization process [62]. On the other hand, a decrease in engraftment of normal murine BM cells in C3- and C5-deficient mice [60, 61] is related similarly as in the case of purinergic signaling to impaired induction of sterile inflammation in recipient BM after myeloablative conditioning of microenvironment. Induction of sterile inflammation in BM facilitates the homing/engraftment of transplanted HSPCs. To explain this phenomenon at the molecular level, C3a and C5a by inducing sterile inflammation in BM and upregulate expression of both chemoattractants for HSPCs (e.g., SDF-1), and adhesion molecules for these cells at the endothelial-BM barrier involved in attachment of transplanted cells navigating from PB to BM [60–62].

#### The Role of Intracellular Complement (complosome) in HSPCs

The recent discoveries on the presence of complosome [14] and pattern recognition receptor Nlrp3 inflammasome [63, 64] in HSPCs shed new light on the role of innate immunity in hematopoiesis. Functional complosome, as mentioned, was initially identified by Kemper et al. in lymphocytes to orchestrate T cell responses and metabolism [11, 12, 51]. Since hematopoiesis and lymphopoiesis have a common hemato/lymphopoietic stem cell origin, we asked if complosome is also expressed and functional in HSPCs. We noticed that human umbilical cord blood (UCB) purified CD34^+^CD38^−^ cells as well as murine Sca-1^+^lin^−^CD45^+^ cells, which both are enriched for a population of hematopoietic stem/progenitor cells (HSPCs) express mRNA for complosome components including C3, C5, C3aR, C5aR1, and C5aR2 [14]. In addition, we found expression of complosome in BM stroma cells upregulated after exposure to G-CSF or Plerixafor during mobilization and in BM stroma conditioned for transplantation by irradiation [14].

Based on this, we focused on the role of the distal part of complosome activation due to C5 cleavage to C5a and employed as a model C5-KO and C5aR-KO mice. The reason for this was our observation that HSPCs isolated from C5-KO mice, in contrast to C3-KO mice [60], showed defects in homing and engraftment in lethally irradiated wild-type animals [14]. This observation implicated intrinsic stem cells defects in C5-KO cells [14]. We reported that HSPCs purified from C5-KO and C5aR-KO mice displayed defective chemotaxis and adhesion and decreased fatty acid, glucose, and amino acid metabolism [14]. The number of BM-stem cells, including HSPCs, was also reduced in C5-KO animals [14]. The defects seen in HSPCs from C5-KO and C5aR-KO mice confirmed that complosome is expressed and functional in normal HSPCs.

Nevertheless, it still needs to be clarified how C5 activation occurs. It could become activated due to the activation of the proximal part of the complosome beginning at the C3 cleavage that forms C5-convertase [11, 12]. Alternatively, the potential activators of C5 in HSPCs, similarly as observed in lymphocytes, could be cathepsin L [11], in tumor cells cathepsin D [57], and in monocytes factor B [65]. This requires further studies, and at this moment, we cannot exclude other potential activators and even simultaneous activation of complementary redundant proteolytic enzyme pathways. Further, work remains to evaluate in more detail the role of the proximal part of the complosome system in regulating the biology of HSPCs and conditioning the BM microenvironment for transplantation. As mentioned, based on Kemper et al. data that provided seminal observations in T lymphocytes, the proximal part of complosome involving C3 cleavage may also as we envision regulate HSPCs biology and this requires further detailed studies [11, 12, 51].

### Coordinated Purinergic Signaling and ComC Activation in Hematopoiesis and their Effect on Intracellular Redox State

**Fig. 2:**
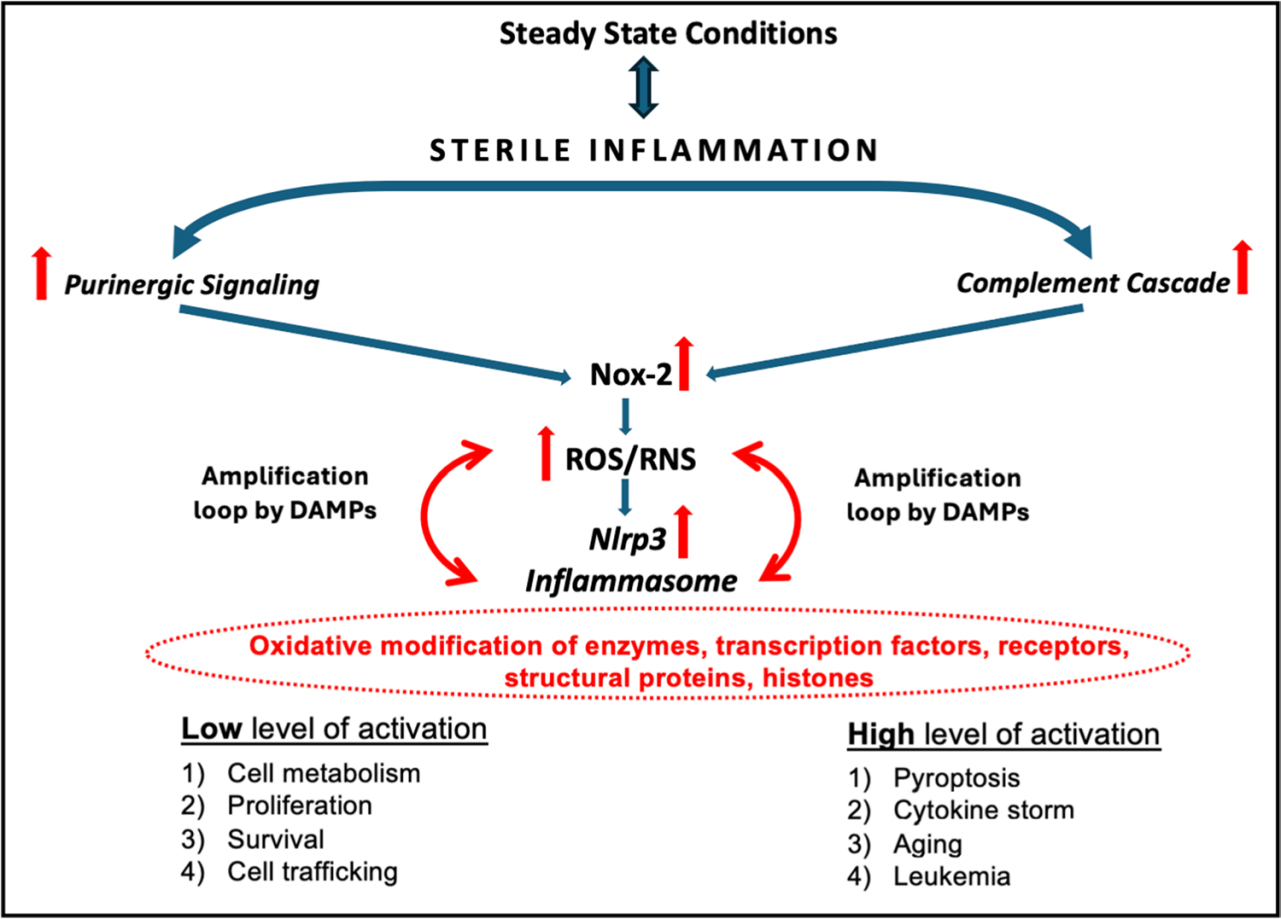
The interplay between purinergic signaling and innate immunity in regulating HSPCs biology - Nlrp3 inflammasome takes central stage. Complement cascade and purinergic signaling regulate sterile inflammation responses in the bone marrow that are essential for the metabolism, proliferation, and trafficking of HSPCs. A significant result of this mutual interaction is the activation of the Nox-2-ROS/RNS-Nlrp3 inflammasome axis that, depending on the activation level, has beneficial or detrimental effects on HSPCs. Nlrp3 inflammasome may modulate several biological processes by secretion of active interleukin-1 beta and interleukin-18 and release from the cells DAMPs, including eATP, HMGB-1, and S100A8 and S100A9 proteins. What we envision is that these DAMPs may, in an autocrine manner, through their Nox-2-associated cell surface receptors, modulate further redox state of the cells (amplification loop) to provide more ROS/RNS that are important signaling mediators because they reversibly oxidize redox-sensitive cysteine and methionine residues present within numerous transcription factors, enzymes, and structural proteins. We postulate that this is a development ancient signaling system still operating, e.g., in HSPCs

Purinergic signaling and innate immunity induce, as discussed above, state of the sterile inflammation in the BM microenvironment during pharmacological mobilization and conditioning for hematopoietic transplantation by radio/chemotherapy [7, 8, 36]. Moreover, these ancient signaling pathways mutually activate each other to potentiate biological effects [28] (Fig. 2). As discussed above, the biological effects of potentially harmful stimuli are usually biphasic: while a low dose can be beneficial to cells, in contrast, a high dose can be damaging. Therefore, the effect of purinergic and ComC signaling in HSPCs depends on the activation level and is positive if it occurs within the beneficial “hormetic zone.” It explains why, under steady-state conditions, low activation of purinergic signaling and innate immunity contribute to maintaining the pool of stem cells in BM (Fig. 2). On the contrary, overactivation could be damaging to cells and lead to death by pyroptosis [8].

#### Effect of Purinergic Signaling and Circulating ComC on Cell Proliferation and Survival

The question remains whether eATP and liver-derived complement may directly stimulate the proliferation of HSPCs. It has been reported, for example, that eATP induces the proliferation of murine embryonic stem cells through PKC, PI3K/Akt, and MAPK signaling pathways engaging P2 receptors [66]. Interestingly, eATP also indirectly affected cell proliferation by modifying the proteome of extracellular vesicles released from microglia [67]. Moreover, eATP also stimulated in a dose- and time-dependent manner the proliferation of breast cancer cell line MCF-7 by phosphorylation of Akt [68]. In contrast, eATP, if added in vitro to murine BM cells, was able to reduce the percentage of common myeloid progenitors and granulocyte–macrophag progenitors, whereas it did not affect differentiation of megakaryocyte–erythroid progenitors [69]. In addition, eATP, if injected into mice, impaired surprisingly BM reconstitution of sublethal irradiated animals [69]. This somehow controversial data could depend on the type of target cells and, most importantly, on doses of eATP employed. To support this, while eATP at low doses enhanced the proliferation and differentiation of dental pulp cells, at higher doses, the effect was the opposite, and this dose dependence supports the concept of hormesis [70]. Our data indicates that eATP does not enhance in vitro growth of HSPCs in clonogenic assays. However, we cannot exclude an intracrine effect of purinergic signaling on the proliferation of these cells [14]. One of the leading in the field group reported that eATP and, to a greater extent, eUTP strongly enhanced the stimulatory activity of several cytokines if employed at suboptimal doses on clonogenic CD34^+^ HSPCs and expanded CD34^+^ cells-derived long-term culture-initiating cells (LTCiC) [71]. Furthermore, this group reported that short-term preincubation of human BM cells before transplantation with UTP significantly expanded the number of marrow-repopulating HSPCs in nonobese diabetic/severe combined immunodeficiency mice [71]. Thus, further investigation is required to assess the role of extrinsic purinergic signaling versus intrinsic one on HSPCs proliferation. As mentioned above, since intracellular ATP may activate the P2X7 receptor expressed on mitochondria [24], by analogy to complosome, an intrinsic purinergic signaling pathway could play as “purinosignalosome” an intracrine role in hematopoiesis. Moreover, P2X7 receptors are also present in other intracellular organelles [48]. It would also be important to investigate if mitochondria express in addition to P2X7 other purinergic receptors.

Similarly, in addition to eATP, circulating in PB liver-derived ComC on cell proliferation needs reappraisals. Because these cells express active complosome, the effect of externally added ComC cleavage fragments as stimulators in clonogenic in vitro assays could be masked by activated intrinsic complosome. Thus, the intracellular activation of ComC in the cytosol may render HSPCs cells independent from stimulation by exogenously applied C3a or C5a. Our recent data indicates that intracellular activation of complosome may play an important role as the number of HSPCs in BM of C5-KO mice is reduced in steady state conditions by ~ 20–30% [14]. Moreover, mice bearing C5-KO HSPCs show delayed recovery of leucocyte and platelet counts from sub-lethal irradiation [14].

**Molecular basis of complosome and “purinosignalosome” mediated effects in HSPCs—the ROS/RNS/Nlrp3 inflammasome signaling axis takes central stage**. As mentioned above, the role of the proximal part of complosome activation has been described in detail in the homeostasis and metabolism of human CD4^+^ lymphocytes [12]. In these cells, C3 is cleaved by cathepsin L to C3a and C3b, and C3a activates C3aR on lysosomes to sustain a low level of mechanistic target of rapamycin (mTOR) for homeostatic survival of these cells [11, 12]. Next, upon activation of T cell receptors, C3a and C3b are secreted from human T cells to engage cell surface C3aR and costimulatory complement receptor CD46 to regulate the production of interferon-gamma to induce human T helper type 1 (Th1) responses [11, 12]. These observations obtained for T human and murine lymphocytes HSPCs need further verification in HSPCs. It would be important to identify the mechanism involved in activating the proximal part of complosome in HSPCs and their impact on their biology. Nevertheless, murine cells, in contrast to human cells, lack CD46 expression, and therefore we can expect some differences between human and murine cells.

In contrast to the activation of the C3 proximal part of the complosome [11, 12], more detailed data on the role of the C5-mediated distal pathway of the complosome in regulating cell proliferation and metabolism still needed to be investigated. Therefore, we became interested in this topic and found that C5-KO or C5aR1-KO mice have defects in the expression of glucose-6 phosphatase dehydrogenase (G6PD) limiting enzyme regulating pentose phosphate cycle required for i) synthesis of NADPH essential for ROS and RNS generation as well ii) synthesis of cholesterol and lipids [14]. In addition, we noticed that mice that lack C5 and C5aR expression also have a decrease in the expression of enzymes involved in lipogenesis, amino acid, and glucose transport [14]. This correlated with a poor activation of Nlrp3 inflammasome. Of note, activation of cell surface C5aR1 by C5a regulates fusion and fission of mitochondria [72]; however, C5aR1, like the P2X7 receptor being expressed in mitochondria itself [11, 12, 65], contributes to ROS release from these organelles and may activate Nlrp3 inflammasome directly.

At the same time, our knowledge of the role of purinosignalosomes in HSPC proliferation is somehow limited and sometimes even controversial. In fibroblasts and neural cells, mitochondria expressed P2X7 receptor, as part of purinosignalosome, regulates mitochondrial respiratory rates, resting potential, level of mitochondria matrix Ca^2+^, and expression of oxidative phosphorylation (OXPHOS) enzymes [24]. In more detail, P2X7 signaling in these cells supports OXPHOS, e.g., by increasing mitochondrial potential and matrix Ca^2+^, which stimulates mitochondrial dehydrogenase and increases the mitochondrial size and thickness [24]. This has a direct effect on cell energy metabolism. Further investigations are required to determine if this phenomenon also occurs in HSPCs. Nevertheless, ATP-P2X7 signaling in mitochondria-like cell surface P2X7 receptor activates Nlrp3 inflammasome [19, 24].

Based on this data Nlrp3 inflammasome takes a central stage as a common target activated by cell membrane expressed C3aR and C5aR1 receptors in response to C3a and C5a, and to non-lytic C5b-C9 as well as by P2X purinergic receptors activated by eATP [19]. Overall, this data supports the unique role of intracellular Nlrp3 inflammasome and its activators (C3a, C5a, and eATP) in the metabolism and trafficking of HSPCs. Again, the Nlrp3 inflammasome may also become activated in a complosome-dependent manner as C5aR, like P2X7 is expressed on mitochondria. Its activation triggers are ROS and RNS derived from the activated cell surface receptors or released in a Nox-2-dependent manner from mitochondria [16, 17, 24]. Nlrp3 inflammasome is an intracellular sensor that recognizes the nutrient supply and cell energetic state and responds accordingly [18, 73, 74]. Within the beneficial “hormetic zone,” activation of Nlrp3 inflammasome positively affects cell metabolism, survival, and trafficking. In this regulatory network, eAdo binding to P1 receptors, and C5a metabolite, _desArg_C5a, interacting with C5aR2 have opposite inhibitory effects [41].

It is well known that hyperactivation of Nlrp3 inflammasome is responsible for cell damage and inflammatory cell necrosis or pyroptosis [5, 19]. Moreover, the prolonged hyperactivation of Nlrp3 inflammasome in response to purinergic signaling or ComC cleavage fragments that occur outside the beneficial “hormetic zone” promotes aging and myelodysplasia and eventually plays a role in the pathogenesis of leukemia [8]. One can finally, ask what is the mechanism by which Nlrp3 inflammasome may modulate all these biological processes as this pattern recognition intracellular receptor associated with proteolytic enzyme caspase-1 activates secretion of active interleukin-1 beta and interleukin-18 and promotes the formation of gasdermin pores in cell membrane to release from the cytosol more DAMPs including eATP, HMGB-1 or S100A8 and S100A9 proteins [18, 73, 74]. We envision that these DAMPs released in response to Nlrp3 inflammasome activation may, in an autocrine manner, activate cells to modulate through their Nox-2-associated cell surface receptors and redox the state of the cells to provide more ROS/RNS. The final response will rely on the level of their intracellular expression (Fig. 2).

To support this, both ROS and RNS were recently identified, as mentioned above, as necessary signaling mediators because they reversibly oxidize redox-sensitive cysteine and methionine residues present within numerous transcription factors, enzymes, and structural proteins [15–17]. These ROS-mediated oxidative post-translational modifications of the target proteins control enzymes' transcription, expression, and biological activity. They also arrange the cellular localization of proteins and/or their interactions with binding partners [15–17]. Examples of ROS-mediated “redox signaling” is a modification of expression and activity of some metabolic enzymes and transcription factors, including AKT kinases, CD39 and CD73 ectonucleotidases, NRF2, HIF-1α, FOXOs, AP1, PTEN, SIRT1 [17, 18]. Nevertheless, despite some progress, the interplay of Nox-2 modulated redox signaling pathways associated with metabolism is still far from fully understood.

Finally, our most recent data indicates that while eATP strongly upregulates the expression of complosome mRNA in HSPCs, Ado has the opposite effect. This supports additional interplay between ancient primordial signaling systems at the extracellular and intracellular levels [14, 58].

**The membrane lipid rafts (MLRs) – mediated effects of purinergic signaling and ComC on orchestrating HSPCs proliferation.** In addition to activating ROS/RNS-Nlrp3 inflammasome signaling, both purinergic signaling and ComC cleavage products may affect the biology of HSPCs by promoting membrane lipid raft (MLRs) formation. MLRs are microdomains that float freely in the membrane bilayer and are enriched for their functional integrity for cholesterol, sphingolipids, and ceramides [8, 75–78]. MLRs incorporate some “raftophilic” receptors involved in hematopoiesis, e.g., the CXCR4 receptor for stromal-derived factor 1 (SDF-1), common β-subunit chain for IL-3, GM-CSF and IL-5 receptors, the c-kit receptor for stem cell factor (SCF), and the VLA-4 integrin receptor [75]. Incorporation of these receptors into MLRs optimizes their signaling to regulate migration, proliferation, and adhesion of HSPCs [8, 75–78].

To explain the role of purinergic and ComC signaling in MLRs formation both these pathways activate the pentose phosphate cycle that provides NADPH for the synthesis of MLRs cholesterol and lipid components [14]. HSPCs defective in C5, C5aR, Nox-2, and P2X7, as well as in Nlrp3 inflammasome, have defects in pentose phosphate required for proper lipogenesis and thus show a decrease in the formation of MLRs [14, 78]. This impairs the signaling from this cell surface membrane expressed “raftophilic receptors.” In addition, in enhancing the synthesis of MLRs lipid components, C3a, C5a, and eATP also play an important role as so-called “priming factors” that facilitate the incorporation of “raftophilic” receptors into MLRs [8, 75–78]. In the presence of these factors, as demonstrated, for example, in the case of CXCR4, this important receptor, if it is incorporated into MLRs, enhances the responsiveness of HSPCs to SDF-1, which is a crucial homing and BM retention chemokine [79]. Similar effects occur for other “raftophilic” receptors and their specific ligands.

## Conclusions

The coordinated action of purinergic signaling and the ComC are early development primordial signaling systems regulating the biology of hematopoietic cells. Both pathways respond to stressors, cross-activate each other, and induce in a Nox2-dependent manner ROS/RNS to activate NLRP3 inflammasome. ROS/RNS emerge as important modifiers of several enzymes, transcription factors, and structural proteins due to oxidation of cysteine/methionine amino acids present in these proteins. Depending on the activation level, activating this network may benefit or harm HSPCs [8, 80]. Currently, small molecular modifiers of purinergic signaling and ComC pathways and their downstream effectors that allow control of these effects are available.

Further work is needed to assess, in addition to Nox-2, the role of other enzymes from the Nox family and antioxidant enzymes in regulating hematopoiesis. Similarly, we need to shed more light on the role of P2Y purinergic receptors. Finally, we must elucidate how the redox state via ROS/RNS regulates the expression of other “classic” hematopoietic mediators, including growth factors, cytokines, chemokines, and bioactive lipids. Modulation of both primordial signaling pathways has important implications for understanding better trafficking of HSPCs and may help to optimize transplantation protocols in clinical hematology. We also need to confirm that the mechanisms described herein for hematopoiesis are universal for other non-hematopoietic tissues.

## Data Availability

Detailed data are available upon reasonable request.
